# Multidimensional Profiles of Recovery: Using Correspondence Analysis to Visualize Physiotherapy Outcomes in Patients with Chronic Low Back Pain

**DOI:** 10.3390/jcm15062305

**Published:** 2026-03-18

**Authors:** Peter Kokol, Helena Blažun Vošner, Jernej Završnik, Alen Pavlec, Urška Šajnović

**Affiliations:** 1 Community Healthcare Center Dr. Adolf Drolc Maribor, 2000 Maribor, Slovenia; helena.blazun@zd-mb.si (H.B.V.); jernej.zavrsnik@zd-mb.si (J.Z.); alen.pavlec@zd-mb.si (A.P.);; 2 Faculty of Electrical Engineering and Computer Sciences, University of Maribor, 2000 Maribor, Slovenia; 3 Health Sciences and Digital Health Transformation Research Unit, University Alma Mater Europaea, 2000 Maribor, Slovenia

**Keywords:** low back pain, physiotherapy, quality of life, stress, multiple correspondence analysis

## Abstract

**Background:** This longitudinal study examined the clinical outcomes of physiotherapy interventions in patients with chronic low back pain, specifically observing the interactions between demographic characteristics, physical metrics, and psychological variables. **Methods:** A cohort of *n* = 150 patients, Final *n* = 123 (18% attrition rate), was assessed using a one-group pre-test/post-test design, with primary outcome measures including Health-Related Quality of Life, the Perceived Stress Scale, and the Numerical Pain Rating Scale. Participants received eight standardized sessions over 4 weeks, including electro-physical agents combined with individualized kinesiotherapy. Data analysis/synthesis was performed via Multiple Correspondence Analysis (MCA) to map associations between categorical variables and treatment responses. **Results:** The predominant clinical profile found was a middle-aged female with moderate educational attainment, presenting with a Body Mass Index in the overweight range and moderate-to-high baseline pain intensity. MCA revealed distinct phenotypic trends: longer Work Experience was associated with lower baseline Quality of Life (QoL) and heightened stress/pain levels. In contrast, patients characterized by higher education and significant Work Experience demonstrated notable post-intervention QoL gains. High baseline QoL served as a predictor for sustained improvement and pain attenuation, while elevated pre-intervention pain scores were consistently linked to perceived unmet clinical needs and exacerbated stress. **Conclusions:** MCA successfully mapped non-linear clusters—such as the “Socio-Psychological Barrier” profile—that traditional univariate methods fail to visualize, suggesting that “individualized care” must prioritize health literacy among patients experiencing extensive work-related strain.

## 1. Introduction

Chronic low back pain (CLBP) is defined as pain or discomfort in the lower back that persists for at least three months and has occurred for at least three days within the past six months. The World Health Organization [1] has identified it as a leading cause of disability, reduced productivity, and diminished quality of life. CLBP is a subjective symptom and can result from various known or unknown deformities or diseases [2]. Demographic factors may also play a significant role [3] and include gender, race, Age, BMI, employment status, income, disability status, and others [4]. While demographic factors like Age and BMI are known contributors, CLBP is fundamentally a biopsychosocial phenomenon [5].

The role of physiotherapy in managing CLBP is well-established. Evidence-based interventions are utilized to address the multifaceted nature of CLBP by alleviating pain and other associated symptoms. These therapeutic approaches contribute to improvements in functional capacity, increased physical activity, and enhanced social engagement. Collectively, these outcomes lead to significant improvements in physical well-being and overall Quality of Life (QoL) in patients [6,7]. A physiotherapist’s verbal and nonverbal interactions with patients, together with their knowledge and skills, directly and indirectly contribute to therapeutic outcomes [8,9,10]. Despite the recent trends in integrating physiotherapy with related disciplines like kinesiology and other healthcare fields, including psychotherapy, physiotherapeutic interventions are still at the cornerstone of successful CLBP rehabilitation [11,12,13].

Various literature reviews suggest that multiple outcome measures are recommended for assessing physiotherapy effectiveness. QoL assessment using the SF-36v2 is highlighted as one of the fundamental factors in CLBP research [14]. QoL manifests across multiple dimensions and highly impacts the success of CLBP therapy [15]. In addition to pathophysiological factors, other elements like depression, anxiety, pain-coping behaviours, and catastrophizing can influence pain intensity, disability, and QoL in patients with CLBP [16].

It is surprising to note that most of the studies analysing the associations between QoL and CLBP are more than 10 years old [17,18], and that they primarily focused on the physical aspects of QoL in relation to CLBP, neglecting psychological, social, and emotional factors [19,20]. To address this gap in knowledge, a longitudinal study was conducted on a randomly selected cohort of patients suffering from CLBP. The participants underwent a standard eight-session intervention of physiotherapeutic rehabilitation at a healthcare institution in Slovenia.

Most existing research relies on univariate or bivariate analyses (e.g., *t*-tests or simple regressions), which isolate variables and fail to capture how they intersect within a single patient. To address this, we employ Multiple Correspondence Analysis (MCA). MCA fills a critical research gap by providing a topological map of patient profiles, allowing us to observe how categorical “social” variables (education) cluster with “clinical” outcomes (pain reduction). An important contribution of this study lies in the application of MCA to identify previously unexplored associations within the context of chronic low back pain. Furthermore, this study demonstrates the utility of MCA as a robust qualitative visualization tool. Finally, it addresses a methodological gap by introducing MCA to physiotherapy research—a field where this analytical approach remains significantly underutilized. Namely, a systematic search of the Web of Science Core Collection was conducted on 6 November 2025 using the Boolean string: TS = (“chronic low back pain” OR “CLBP”) AND TS = (“multiple correspondence analysis” or MCA). No year restriction was applied. The search resulted in only two publications, published in 2009 and 2017. Lefevre-Colau et al. reported on the interrelations of risk factors in CLBP [21], and Bailly et al. reported on the validation of a questionnaire about behavioural strategies in CLBP [22].

## 2. Methodology

Participants for the presented longitudinal study were randomly sampled from the population of patients with CLBP undergoing a rehabilitation program performed by the Physiotherapy Department of a Slovenian public healthcare institution. Study participants were randomly selected using a sampling ratio of six to one—meaning that among each of six eligible patients, one was included in this study. The minimum sample size calculation using the G*Power program (version 3.1.9.7) (Heinrich Heine University Dusseldorf, Germany) multiple-variable analysis (10 variables, effect size = 0.1, error probability = 0.05, Power = 0.95) resulted in a cut-off value of 110 participants. The inclusion criteria were as follows: Age between 18 and 75 years (inclusive), the presence of chronic low back pain persisting for more than three months [23,24], and completion of physiotherapy treatment for CLBP. While this study aimed to maintain high ecological validity, patients were screened for ‘red flags’ during the initial functional assessment. Standard contraindications for the electro-physical agents used (e.g., active malignancy, pregnancy, or implanted electronic devices) served as implicit exclusion criteria. The study period was from July 2024 to June 2025.

During the preparation of this work, the authors used Gemini 3 for spelling and grammar checking. After using this tool/service, the authors reviewed and edited the content as needed and took full responsibility for the final content of the publication.

### 2.1. Measurement Instruments

The Quality of life (QoL) was measured with the 36-Item Short Form Survey Instrument (SF-36v2) [25]. Overall QoL was calculated as the average of all eight dimensions. The higher score means higher QoL. Perceived stress was assessed with the Perceived Stress Scale (PSS) [26], and the intensity of CLBP was measured with the paper-based Numerical Pain Rating Scale (NPRS) [27]. Participants completed a demographic questionnaire specifically developed for this study (sex, Age, height, weight, educational attainment, total years of employment, and satisfaction with monthly income) with the assistance of the principal investigator. The fulfilment of needs was assessed using a single-item Numerical Rating Scale. Participants were asked to rate their overall level of need fulfilment on a scale ranging from 1 (‘not at all fulfilled’) to 10 (‘completely fulfilled’). This approach is consistent with Self-Determination Theory. For this study, the SF-36v2 and PSS were translated from English into Slovenian by a medical specialist and subsequently back-translated into English by a public health professional to ensure conceptual and linguistic equivalence. Both experts also compared the translated versions with existing Slovenian versions of the SF-36 questionnaire [28] and the PSS questionnaire [29] and performed the necessary adaptations. Finally, all questionnaires were pilot tested on a sample of 10 patients. Data were measured before and after the intervention, classifying the study design as a One-Group Pre-Test–Post-Test Study.

### 2.2. Therapeutic Protocol

The physiotherapy intervention comprised eight sessions, aligned with standard Slovenian clinical referral patterns and rehabilitation protocols. Following an initial medical examination, a physiotherapist conducted a functional assessment to design a multimodal, individualized treatment plan. The therapeutic modalities utilized within this protocol are summarized in Table 1. The integration of these techniques aimed to provide a holistic approach to patient recovery by addressing both physiological symptoms and long-term activity management.

### 2.3. Statistical Analysis

Multiple Correspondence Analysis, a very useful but underutilized method [30], was used to visualize associations between variables and perform the analysis. MCA is a multivariate statistical and visualization method designed to analyse multiway contingency tables, which reflect associations between rows and columns. MCA produces intuitive biplots that display the associations between categories of categorical variables, with closer items indicating stronger associations. These biplots consist of two dimensions, which are labelled during the analysis, adding a qualitative dimension to the results [30]. The analysis was performed using SPSS software V28 (IBM, Rochester), applying Optimal scaling. Outlier detection was not applied; however, the data were administratively checked for punctuation, lexical errors, and out-of-range/implausible values. Continuous variables were transformed into categorical variables using the SPSS discretization procedure. After several iterations with various binning techniques and sizes, Equal-frequency binning into five categories per variable was selected as the most optimal option for visual interpretation of the joint plot. Binning threshold values are shown in Table 2.

The joint plot was interpreted in a qualitative way [31,32]. Both studies demonstrate the utility of MCA, one even within the field of physiotherapy, for visualizing associations between categorical variable. While comprehensive statistical results have been detailed in previous literature [7], this paper focuses on the qualitative interpretation of these relationships.

## 3. Results

A total of 150 participants were initially enrolled in this study, and 27 (18%) withdrew during this study, resulting in a final sample of 123 participants. A bias analysis was conducted comparing the 27 dropouts with the 123 completers; no significant differences were found in baseline NPRS scores (*p* > 0.05) or Age (*p* > 0.05), suggesting that the final sample remained representative. The reasons for withdrawal are not known, as these patients did not show up. Among the remaining 123 participants, there were 94 females and 29 males. Ten participants had completed basic school (9 years of education), 80 had completed high school (12 or 13 years of education), 29 had completed higher education (16 years of education), and four earned a master’s or PhD degree. Central tendency values for other demographic variables are shown in Table 3.

To determine the dimensionality of the correspondence space, the eigenvalues and inertia were analysed (Table 4). Dimension 1 and Dimension 2 yielded eigenvalues of 3.625 and 2.630, respectively. Combined, these two dimensions account for 52.1% of the total inertia in the dataset. This level of explained variance suggests that a two-dimensional solution provides a satisfactory representation of the associations between the categorical variables, capturing most of the underlying structure. In MCA, which handles high-dimensional categorical data, a cumulative inertia is considered a robust representation [33] of the primary structural patterns, particularly in clinical datasets where “noise” is expected.

**Figure 1:**
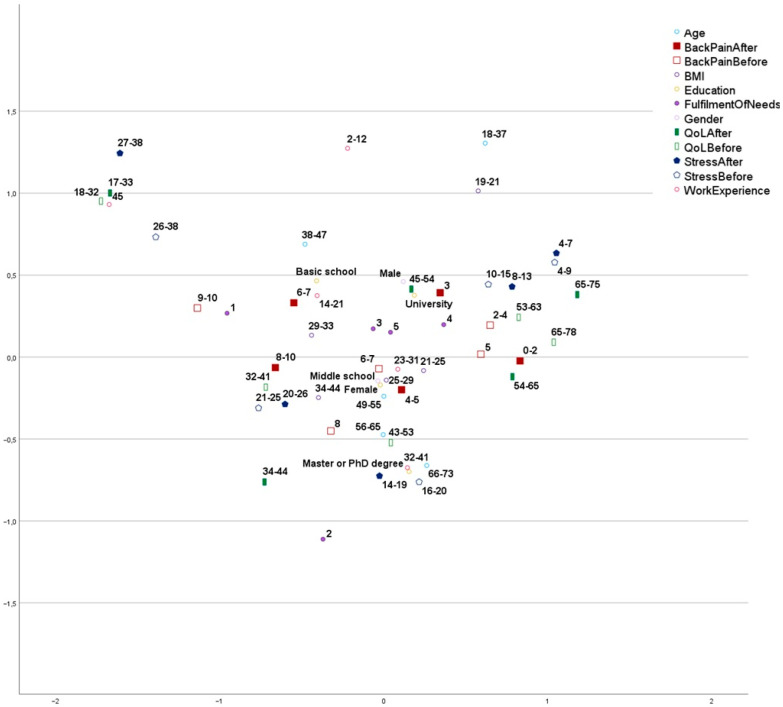
Visualization of associations between variables using MCA.

The analysis of associations in Figure 1 revealed a typical participant profile: female, with a middle school education, aged 49–55, experiencing perceived pain levels of 6–7 before and 4–5 after physiotherapy intervention, a BMI (calculated as Weight/Height^2^ in kg/m^2^) between 21 and 29, and 23–31 years of Work Experience.

**Figure 2:**
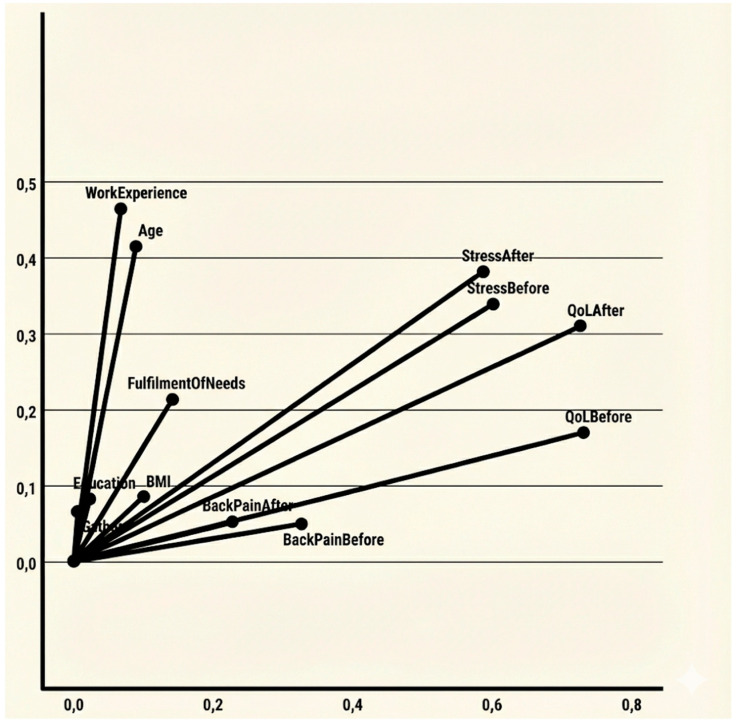
Discrimination measures.

The discrimination measures (Table 5 and Figure 2) reveal the variables that most strongly contribute to the two dimensions.

- **Dimension 1:** This dimension is primarily defined by psychological and health-related indicators. The strongest discriminators are Quality of Life before (0.731) and after (0.726) the intervention, followed by Stress levels before (0.601) and after (0.587). Back pain indicators also contribute moderately to this axis, particularly Back Pain Before (0.327). Dimension 1 can be qualitatively labelled as Well-being and Health Status.
- **Dimension 2:** This dimension is characterized by the life stage and career profile of the participants. The primary drivers are Work Experience (0.465) and Age (0.414). Stress levels after (0.381) and Quality of Life after (0.310) also show secondary associations with this dimension. Dimension 2 can be qualitatively labelled Demographic and Professional Maturity.

Based on the above observations, along with qualitative assessment of variable values and inter-point distances (Figure 1), the following patterns were identified:

- **Longest Work Experience:** Patients in this group exhibited the lowest Quality of Life (QoL) and the highest levels of stress and pain. Following multimodal individualized physiotherapy, QoL, stress, and pain measurements remained largely unchanged. (left upper corner of the plot).
- **Higher Education, Long Work Experience, and Older Age:** These patients initially experienced moderate stress and high pain. After the intervention, QoL measurements for this group were higher than baseline (down middle of the plot).
- **Highest Baseline QoL:** Patients with the highest QoL prior to the intervention maintained the highest QoL post-physiotherapy. Final measurements showed higher QoL levels and lower pain levels compared to initial assessments (middle right of the plot).
- **Highest Pre-Intervention Pain**: The highest pre-intervention pain levels were associated with the lowest fulfilment of needs (middle left of the plot).
- **High Pain and Stress and Low QoL**: High pain was associated with high stress, low QoL, and limited pain reduction (referring to a potential worsening of pain or a less-than-desired level of pain reduction (central part of the plot).

If we limit our interpretation only to points exhibiting high discrimination measures (Table 5), we can discern two clusters:

- Cluster 1 (Vulnerability): There is a clear overlap between “Longest Work Experience” and “Highest Stress”. These points represent a statistically similar space where traditional physiotherapy shows minimal impact on QoL.
- Cluster 2 (Recovery): Higher education and high baseline QoL cluster together, predicting the largest improvements in post-intervention pain.

## 4. Discussion

Our results suggest that recovery is not just a physical process but is tethered to a patient’s Social Capital [34]. The association between education level and recovery supports the Health Literacy Theory [35,36]. Highly educated patients are likely to possess better coping mechanisms and higher self-efficacy, which allows them to integrate physiotherapeutic advice more effectively. Conversely, the cluster of “Long Work Experience” with “High Stress” indicates that chronic occupational strain may create a “ceiling effect”, where physical therapy alone cannot overcome the psychological burden of a demanding career. Our findings align with Kadir et al. [37] regarding work strain but contradict earlier univariate studies that suggested Age as the primary predictor [38].

Using MCA, we demonstrated that education might be a more potent baseline characteristic for QoL improvement than Age. Finally, this study demonstrates that Multiple Correspondence Analysis is a powerful, yet underutilized, tool for uncovering the multidimensional relationships between demographic factors, pain, and Quality of Life in patients with chronic low back pain. By transforming complex data into intuitive bi-plots, MCA facilitated a unique qualitative visualization of patient profiles, revealing previously unexplored associations that traditional statistical methods might overlook. The application of MCA addresses a significant methodological gap in physiotherapy research, offering a robust framework for capturing the multifaceted nature of clinical outcomes and patient recovery.

### 4.1. Ideas for Further Research

This study lays the groundwork for several avenues of future investigation. Future research could delve more deeply into the impact of demographic factors on pain, specifically by incorporating data on patients’ broader socioeconomic status and the psychosocial stressors they experience in both domestic and professional settings. Furthermore, our findings provide a basis for a more in-depth exploration of subjective factors in CLBP, particularly the relationship between emotional well-being and pain.

### 4.2. Implications for Physiotherapy Practice

The findings of this study have potential implications for the clinical management of CLBP. They underscore the importance of physiotherapists adopting a multidimensional, patient-centred approach that addresses not only the physical symptoms but also the emotional and social dimensions of the condition. By considering a patient’s overall Quality of Life, accounting for potential demographic influences, and addressing psychological and emotional factors, practitioners can strive for more comprehensive and meaningful treatment outcomes.

### 4.3. Study Limitations

This study, despite its integration of multiple aspects of QoL, demographic variables, and the application of robust multivariate correspondence analysis, has several limitations. A key limitation is the absence of a precise definition regarding some specifics of CLBP diagnoses, especially the duration of CLBP prior to the commencement of the rehabilitation process.

Furthermore, while the selection of measurement instruments was informed by a review of pertinent scientific literature on CLBP, the choice of assessment tools represents a limitation. The potential for different instruments to produce divergent results suggests that this methodological choice may have influenced the findings.

This study utilized a One-Group Pre-Test–Post-Test Study design with a limited observation period of eight working sessions. According to the study type, all associations must be treated as observed, and the patterns as exploratory. However, while the One-Group design limits causal claims, it is appropriate for this exploratory mapping of multidimensional interactions. Additionally, while a One-Group design cannot exclude placebo effects or natural recovery, it is appropriate for this exploratory phase of mapping multidimensional associations.

Although the sample was predominantly female (76%), MCA is mathematically robust to unbalanced group sizes, as it calculates distances based on relative frequencies. This allows the model to map male-specific associations (e.g., high Work Experience) within the same geometric space without the results being overwhelmed by the larger female cohort.

Finally, the lack of strict exclusion criteria regarding specific CLBP aetiologies (e.g., disc herniation vs. spondylolisthesis) may increase the heterogeneity of the findings.

## 5. Conclusions

This study demonstrates that the clinical management of CLBP requires more than a “one-size-fits-all” physical approach. We conclude that patients with lower health literacy and extensive Work Experience are at high risk for poor outcomes. We recommend that future physiotherapy protocols for these demographic incorporate interventions such as e “Pain Neuroscience Education” to address the psychological clusters identified by the MCA.

## Figures and Tables

**Table 1:** Therapeutic modalities (All sessions were occurred in a temperature-controlled clinical environment (approximately 22 degrees Celsius and were performed by therapists with more than 5 years of experience, maintaining moderate intensity according to the Borg scale).

Category	Therapeutic Modalities
Electro-Physical Agents	15 min of magnetotherapy (mT), ultrasound therapy (1.0 W/cm^2^), laser therapy, TENS, and interferential therapy
Manual and Active Therapy	Manual therapy and individualized kinesiotherapy: 20 min of individualized kinesiotherapy (core stabilization and stretching)

**Table 2:** Binning thresholds used for MCA analysis (*n* = 123).

Variable	Bin 1	Bin 2	Bin 3	Bin 4	Bin 5
Age (years)	18–37	38–47	49–55	56–65	66–73
Back Pain After	0–2	3	4–5	6–7	8–10
Back Pain Before	2–4	5	6–7	8	9–10
BMI (kg/m^2^)	19–21	21–25	25–29	29–33	34–45
Fulfillment of Needs	1	2	3	4	5
QoL After	17–33	33–44	44–54	54–65	56–78
QoL Before	18–32	32–41	43–53	53–63	65–75
Stress After	4–7	8–13	14–19	20–26	27–38
Stress Before	4–9	10–15	16–20	21–25	26–38
Work Experience (Years)	2–12	14–21	23–31	32–41	45

QoL—Quality of Life; BMI—Body Mass Index.

**Table 3:** Central tendency of demographic variables (*n* = 123).

	Minimum	Maximum	Mean	Std. Deviation
Age (Years)	18	73.0	51.6	11.4
BMI (kg/m^2^)	14.5	44.1	27.5	5.5
Work Experience (Years)	2.0	45.0	26.8	11.7

BMI = Body Mass Index.

**Table 4:** Model summary: eigenvalues and inertia decomposition for MCA dimensions.

	Total (Eigenvalue)	Inertia
Dimension 1	3.625	0.302
Dimension 2	2.630	0.219
Total	6.255	0.521

**Table 5:** Discrimination measures.

	Dimension 1	Dimension 2
Gender	0.004	0.067
Age	0.089	0.414
BMI	0.100	0.085
Education	0.023	0.083
Work Experience	0.068	0.465
Fulfilment of Needs	0.142	0.214
Back Pain Before	0.327	0.050
Back Pain After	0.228	0.053
QoL Before	0.731	0.170
Stress Before	0.601	0.339
Stress After	0.587	0.381
QoL After	0.726	0.310

QoL—Quality of Life; BMI—Body Mass Index.

## Data Availability

The data presented in this study are available from the corresponding author upon reasonable request due to the fact that data are not publicly available due to privacy and ethical restrictions.
